# Supplementary material for: Cross-neutralization of a SARS-CoV-2 antibody to a functionally conserved site is mediated by avidity
Source: bioRxiv. 2020 Aug 3:2020.08.02.233536. Preprint. [Version 1] doi: 10.1101/2020.08.02.233536 (PMC7418720; doi:10.1101/2020.08.02.233536)
Supplement: 1 [file NIHPP2020.08.02.233536-supplement-1.pdf]

bioRxiv preprint doi: https://doi.org/10.1101/2020.08.02.233536. this version posted August 3, 2020. The copyright holder for this preprint (which was not certified by peer review) is the author/funder. It is made available under a CC-BY 4.0 International license.

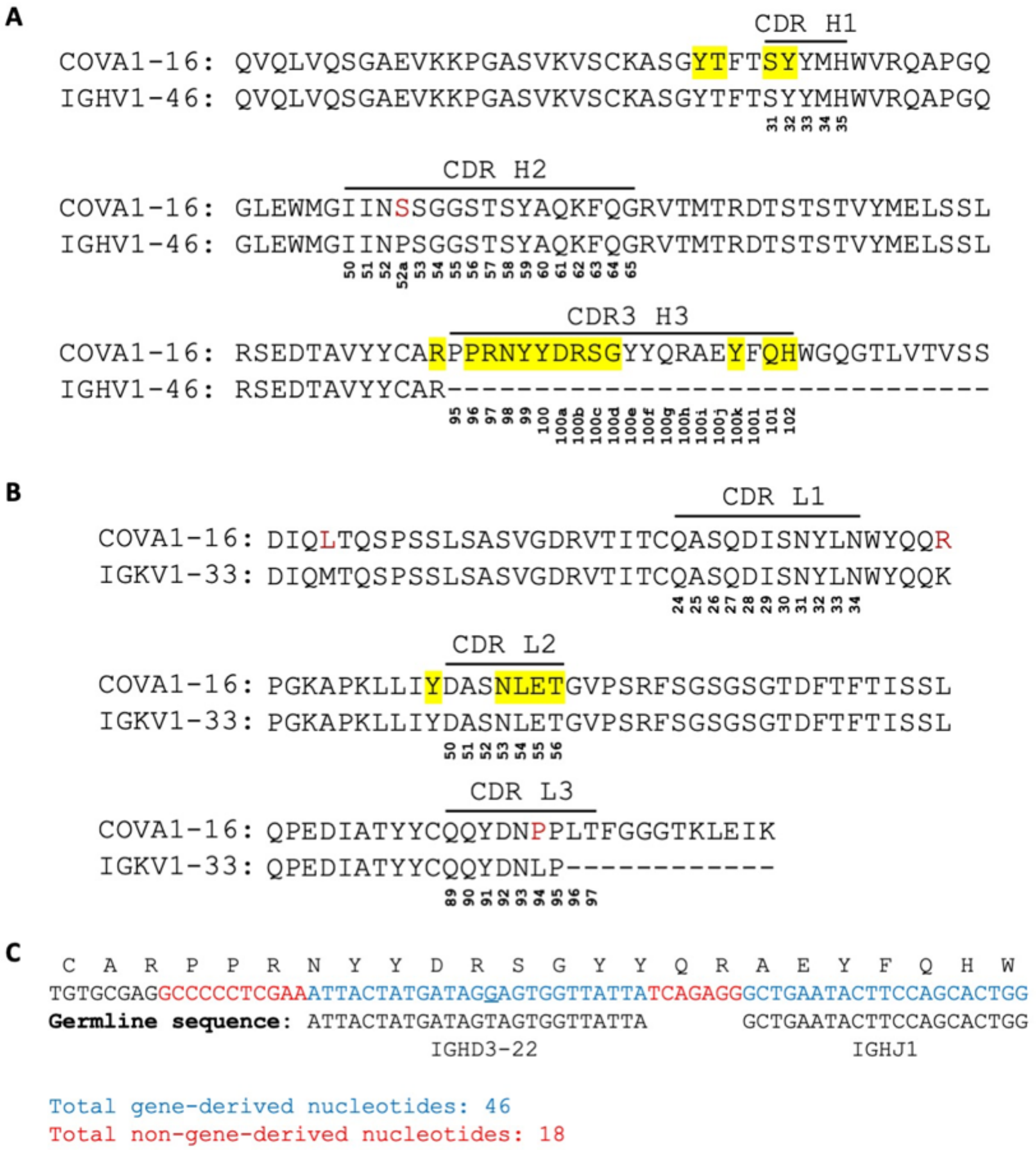

Supplementary Figure 1. Comparison of COVA1-16 and putative germline sequences. Alignment of COVA1-16 Fab amino-acid sequence with **(A)** germline IGHV1-46 sequence, and **(B)** germline IGKV1-33 sequence. The regions that correspond to CDR H1, H2, H3, L1, L2, and L3 are indicated. Residues that differ from germline are highlighted in red. COVA1-16 Fab residues that interact with the RBD are highlighted in yellow. Residue positions in the CDRs are labeled according to the Kabat numbering scheme. **(C)** Amino acid and nucleotide sequences of the V-D-J junction of COVA1-16, with putative

bioRxiv preprint doi: https://doi.org/10.1101/2020.08.02.233536. this version posted August 3, 2020. The copyright holder for this preprint (which was not certified by peer review) is the author/funder. It is made available under a CC-BY 4.0 International license.

gene segments (blue) and N-regions (red), are indicate. The germline sequences of IGHD3-22 and IGHJ1 are also shown. The only somatically mutated nucleotide in the D region is underlined.

bioRxiv preprint doi: https://doi.org/10.1101/2020.08.02.233536. this version posted August 3, 2020. The copyright holder for this preprint (which was not certified by peer review) is the author/funder. It is made available under a CC-BY 4.0 International license.

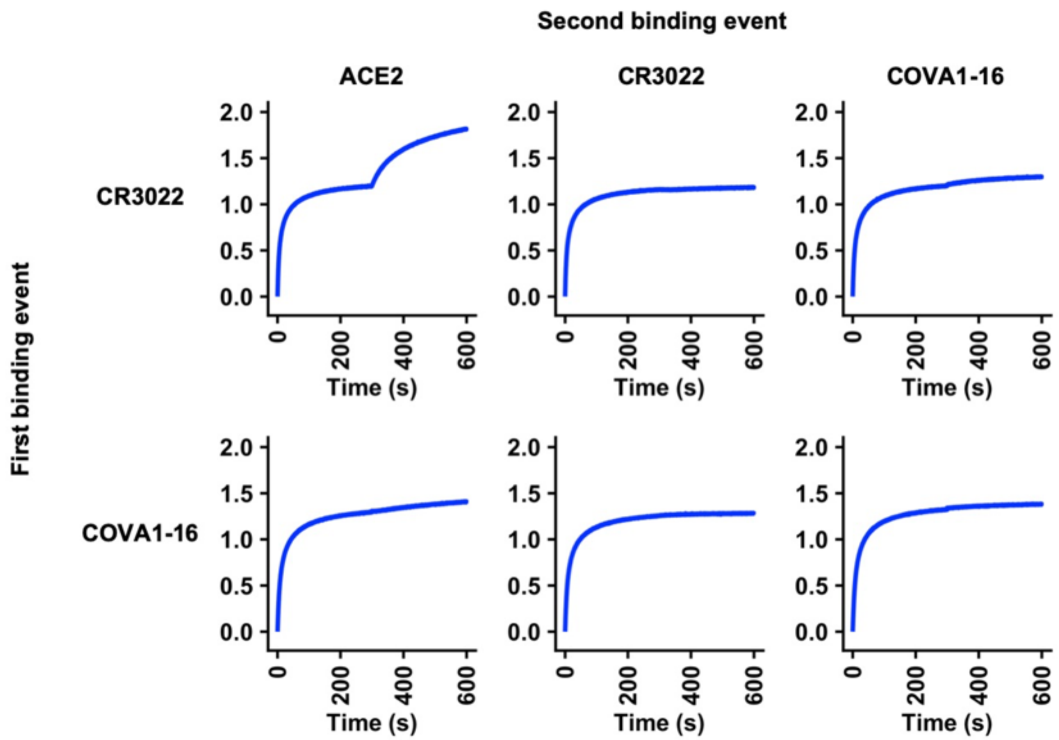

Supplementary Figure 2. Competition assay between different IgGs and ACE2. Competition between COVA1-16 IgG, CR3022 IgG, and Fc-tagged ACE2 was measured by biolayer interferometry (BLI). Y-axis represents the response. The biosensor was first loaded with SARS-CoV-2 RBD, followed by two binding events: 1) CR3022 IgG or COVA1-16 IgG, and 2) ACE2, CR3022 IgG, or COVA1-16 IgG. A period of 300 s was used for each binding event. A further increase in signal during the second binding event (starting at 300 s time point) indicates lack of competition with the first ligand.

bioRxiv preprint doi: https://doi.org/10.1101/2020.08.02.233536. this version posted August 3, 2020. The copyright holder for this preprint (which was not certified by peer review) is the author/funder. It is made available under a CC-BY 4.0 International license.

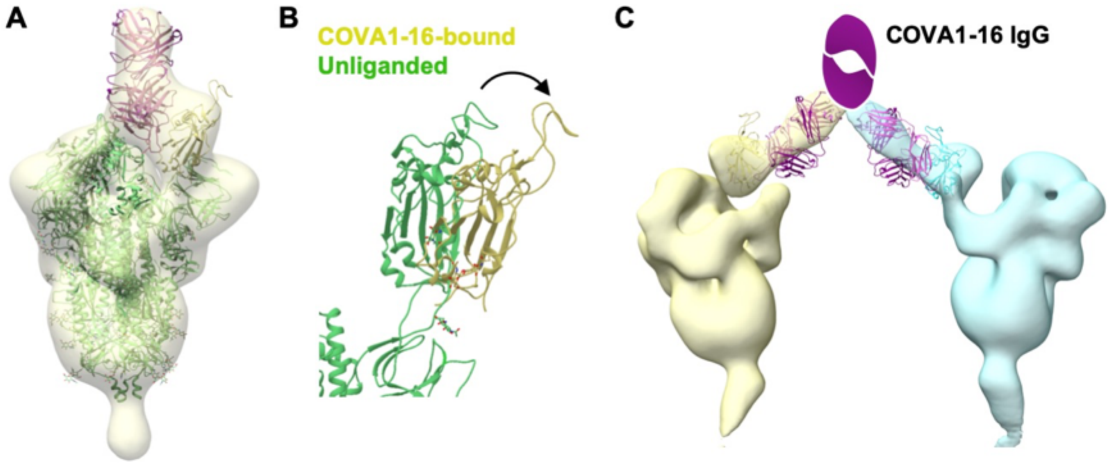

Supplementary Figure 3. Negative-stain EM analysis of COVA1-16 binding to SARS-CoV-2 S trimer. (A) An atomic model from the crystal structure of SARS-CoV-2 RBD bound to COVA1-16 Fab was fit into the negative-stain EM reconstruction of the SARS-CoV-2 spike bound to COVA1-16 Fab. The COVA1-16 Fab approaches the apex of the S trimer in a perpendicular orientation. A secondary structure backbone representation of the prefusion spike model (PDB: 6Z97, green) [1] was also fit into the EM density with RBD residues (334-528) removed from one of the protomers here for clarity. The COVA1-16 heavy and light chains are in magenta and pink, respectively, and COVA1-16-bound RBD in yellow. **(B)** Conformation of RBD in an up conformation from an unliganded SARS-CoV-2 S trimer (PDB: 6Z97, green) [1] is compared to that of the RBD (yellow) bound by COVA1-16 Fab. The arrow indicates that the RBD further rotates and opens up when bound to COVA1-16, thereby moving further away from the trimer threefold axis. **(C)** An atomic model of the spike RBD bound to COVA1-16 Fab is fit into a negative-stain EM reconstruction, where COVA1-16 Fab approaches the SARS-CoV-2 S trimer from the side. COVA1-16 is modelled as an IgG to illustrate the feasibility of bivalent binding to adjacent spike proteins on the virus surface. The Fab heavy and light chains are shown in magenta and pink. A schematic representation of the Fc domain of the IgG is shown in

bioRxiv preprint doi: https://doi.org/10.1101/2020.08.02.233536. this version posted August 3, 2020. The copyright holder for this preprint (which was not certified by peer review) is the author/funder. It is made available under a CC-BY 4.0 International license.

magenta. The RBD model and spike density for each trimer is shown in yellow and cyan.

bioRxiv preprint doi: https://doi.org/10.1101/2020.08.02.233536. this version posted August 3, 2020. The copyright holder for this preprint (which was not certified by peer review) is the author/funder. It is made available under a CC-BY 4.0 International license.

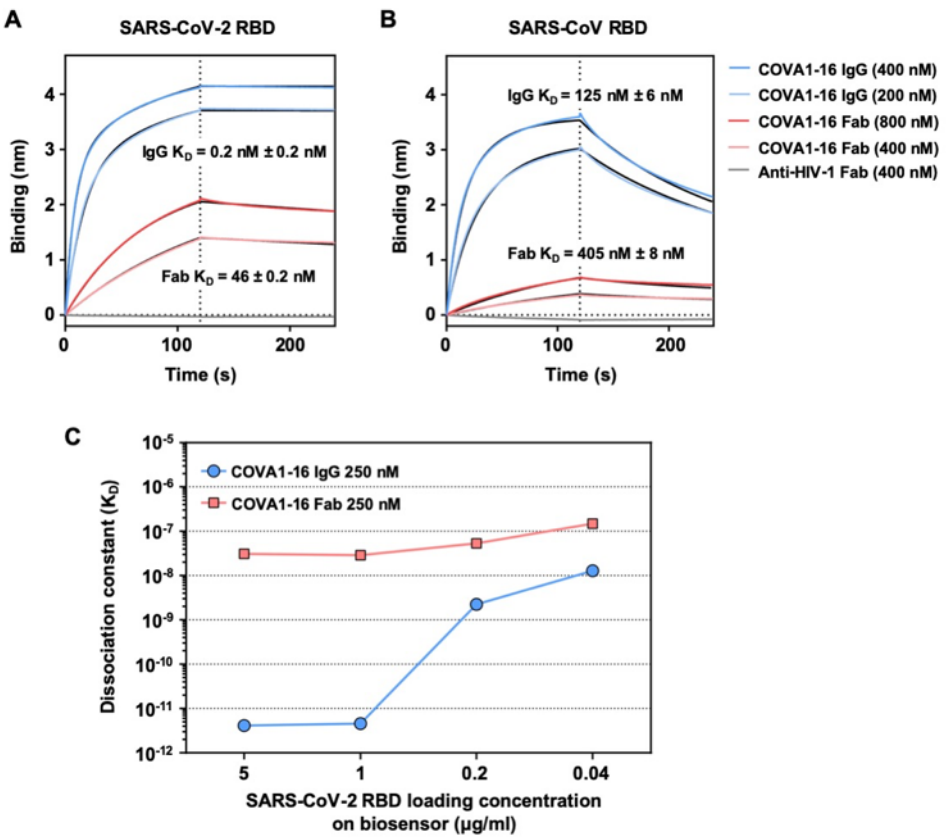

Supplementary Figure 4. Sensorgrams for binding of COVA1-16 to SARS-CoV-2 RBD and SARS-CoV RBD. (A-B) Binding kinetics of COVA1-16 Fab and IgG to **(A)** SARS-CoV-2 RBD and **(B)** SARS-CoV RBD were measured by biolayer interferometry (BLI) with RBD on the biosensor and antibody in solution. Y-axis represents the response. An anti-HIV His-tagged Fab (4E1) was used as a negative control. Dissociation constants (K_D) for IgG and Fab were obtained using a 1:2 bivalent model and 1:1 binding model, respectively, which are represented by the black lines. Representative results of two replicates for each experiment are shown. **(C)** The relationship between SARS-CoV-2 RBD loading concentration on the biosensor and the dissociation constant of COVA1-16 IgG is shown.

bioRxiv preprint doi: https://doi.org/10.1101/2020.08.02.233536. this version posted August 3, 2020. The copyright holder for this preprint (which was not certified by peer review) is the author/funder. It is made available under a CC-BY 4.0 International license.

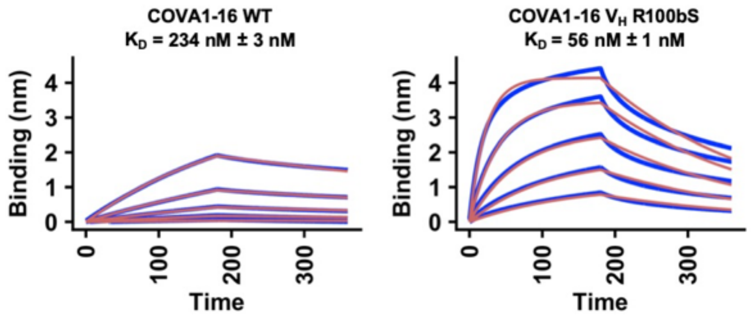

Supplementary Figure 5. Sensorgrams for binding of COVA1-16 wild-type and V_H R100bS mutant Fabs to SARS-CoV-2 RBD. Binding kinetics of COVA1-16 wild-type and V_H R100bS mutant Fab to SARS-CoV-2 RBD were measured by biolayer interferometry (BLI) with RBD on the biosensor and antibody in solution. Y-axis represents the response. Dissociation constants (K_D) for Fabs were obtained using a 1:1 binding model, which are represented by the red lines. Representative results of two replicates for each experiment are shown. Unlike Figure S4, which used HEK293F-expressed SARS-CoV-2, the experiment here used insect cell-expressed SARS-CoV-2.

bioRxiv preprint doi: https://doi.org/10.1101/2020.08.02.233536. this version posted August 3, 2020. The copyright holder for this preprint (which was not certified by peer review) is the author/funder. It is made available under a CC-BY 4.0 International license.

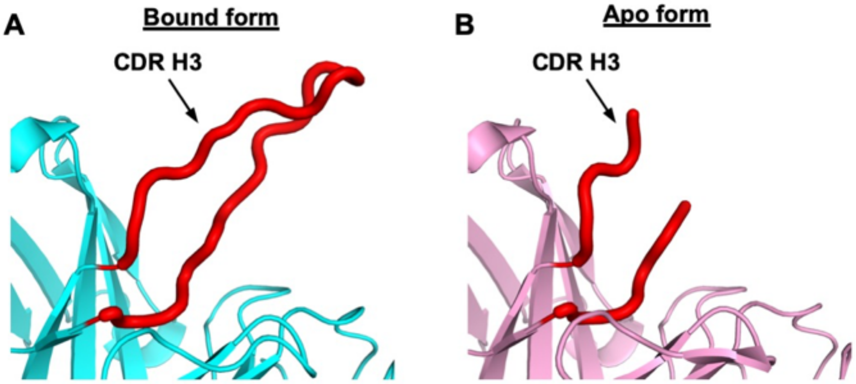

Supplementary Figure 6. CDR H3 of COVA1-16 Fab is disordered in its unliganded apo form. (A) In the crystal structure of the RBD-bound form of COVA1-16 Fab, the CDR H3 loop is completely ordered (red). **(B)** In the crystal structure of the apo form of COVA1-16, the distal end of the CDR H3 loop is intrinsically disordered or flexible (red).

bioRxiv preprint doi: https://doi.org/10.1101/2020.08.02.233536. this version posted August 3, 2020. The copyright holder for this preprint (which was not certified by peer review) is the author/funder. It is made available under a CC-BY 4.0 International license.

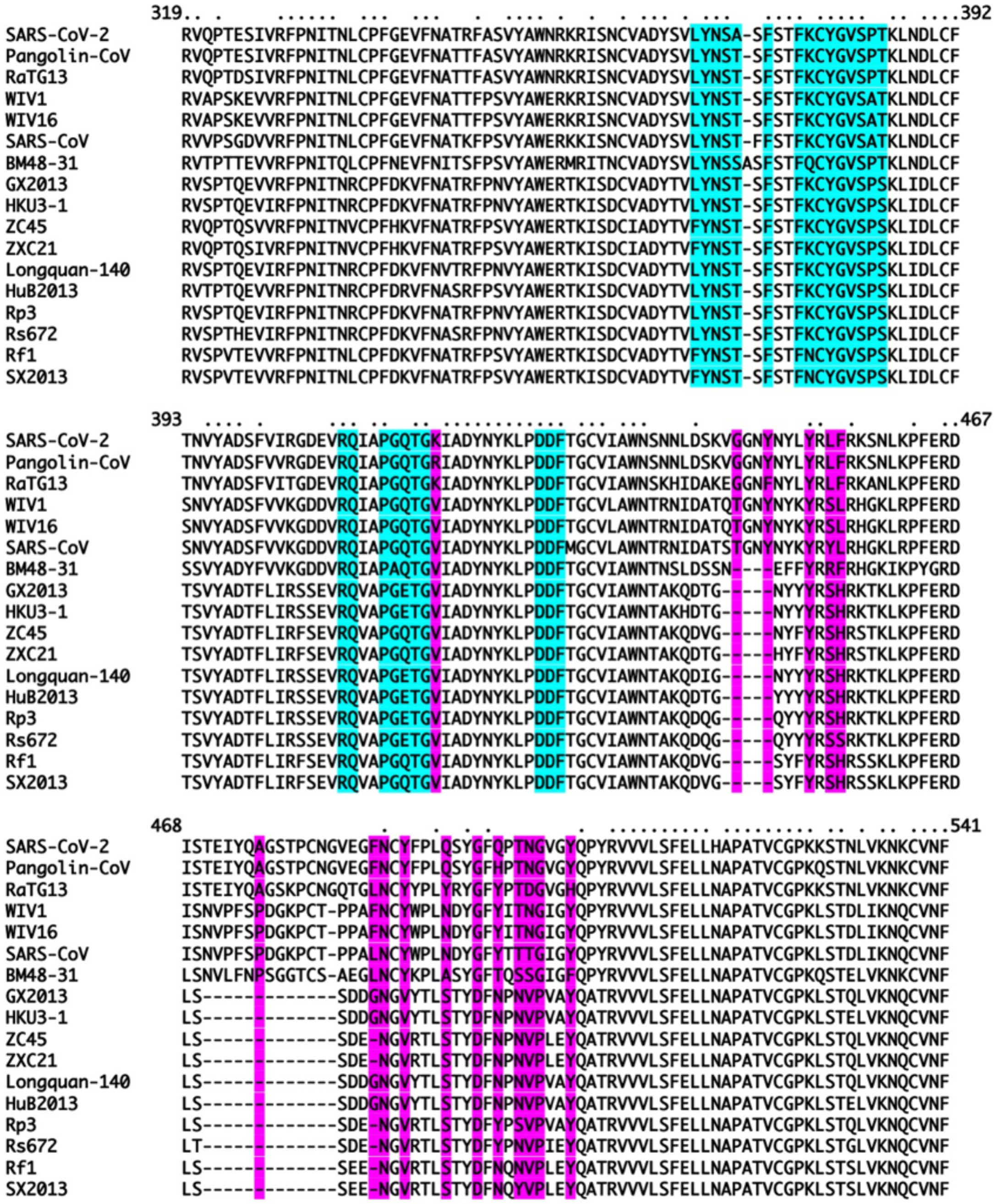

Supplementary Figure 7. Sequence alignment of the RBD from SARS-related coronaviruses. Amino-acid sequences of RBDs from SARS-CoV-2, SARS-CoV, and other SARS-related coronavirus (SARSr-CoV) strains are aligned. COVA1-16 epitope residues are highlighted in cyan. ACE2-binding residues are highlighted in purple. Conserved residues are indicated by small black dots on the top of the alignment.

bioRxiv preprint doi: https://doi.org/10.1101/2020.08.02.233536. this version posted August 3, 2020. The copyright holder for this preprint (which was not certified by peer review) is the author/funder. It is made available under a CC-BY 4.0 International license.

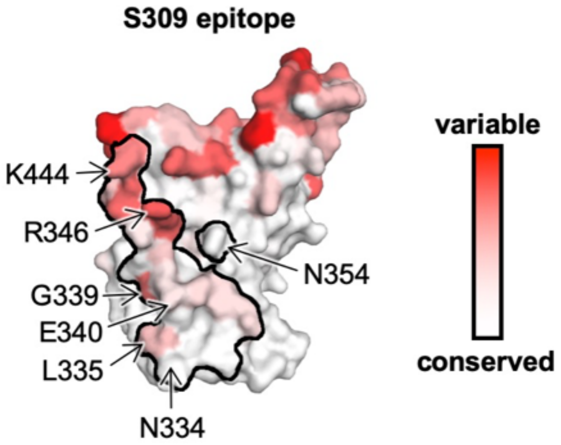

Supplementary Figure 8. Sequence conservation of S309 epitope. Sequence conservation of the RBD is highlighted on the structure for S309 epitope [2]. This view corresponds to the opposite side (rotated 180 degrees along the vertical axis) from that shown in Figure 4A-B.

bioRxiv preprint doi: https://doi.org/10.1101/2020.08.02.233536. this version posted August 3, 2020. The copyright holder for this preprint (which was not certified by peer review) is the author/funder. It is made available under a CC-BY 4.0 International license.

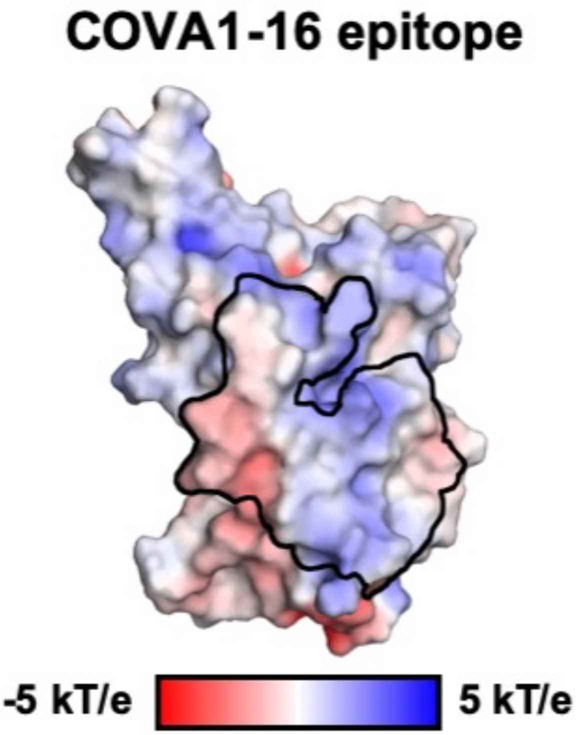

Supplementary Figure 9. COVA1-16 epitope in electrostatic surface representation.

The epitope of COVA1-16 is outlined and shows its largely polar nature.

bioRxiv preprint doi: https://doi.org/10.1101/2020.08.02.233536. this version posted August 3, 2020. The copyright holder for this preprint (which was not certified by peer review) is the author/funder. It is made available under a CC-BY 4.0 International license.

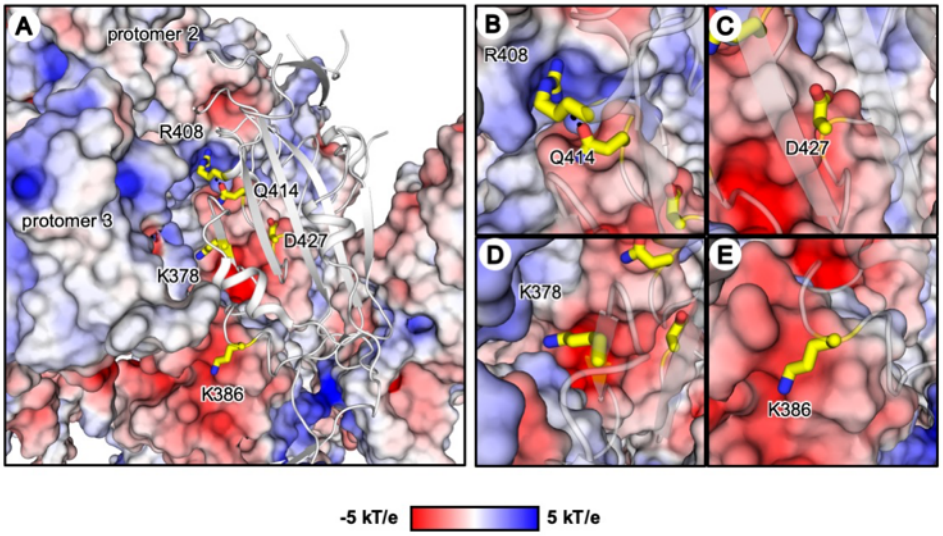

Supplementary Figure 10. Location of residues of interest in the COVA1-16 epitope when all three RBDs are in the "down" conformation. (A) The RBD of one of the three protomers is shown as a gray cartoon with the side chains of five residues of interest shown in yellow stick representation. RBD residues K378, R408, Q414, and D427 are within the COVA1-16 epitope, whereas K386 is not a COVA1-16 epitope residue. The other two protomers (protomers 2 and 3) are shown in a surface electrostatic representation. **(B-E)** Zoomed-in views for the regions surrounding residues **(B)** R408 and Q414, **(C)** D427, **(D)** K378, and **(E)** K386**.** A hydrogen bond in **(B)** is represented by a dashed line. Due to charge difference or similarity between the side chain and the proximal region of the neighboring protomer, either repulsive (same charge) or attractive (opposite charge) environments are found and visualized here. PDB 6VXX is used to represent the spike protein [3]. Of note, the shape complementarity values (Sc) [4] of the COVA1-16 epitope/RBD interface, COVA1-16 epitope/S2 interface, and COVA1-16 epitope/COVA1-16 interface are 0.53, 0.75, and 0.74, respectively, indicating good complementary and tight fit of the COVA1-16 epitope surface with the rest of the trimer in the RBD down

bioRxiv preprint doi: https://doi.org/10.1101/2020.08.02.233536. this version posted August 3, 2020. The copyright holder for this preprint (which was not certified by peer review) is the author/funder. It is made available under a CC-BY 4.0 International license.

conformation. Sc values can range from 0 to 1, with a larger Sc value represents higher shape complementarity.

bioRxiv preprint doi: https://doi.org/10.1101/2020.08.02.233536. this version posted August 3, 2020. The copyright holder for this preprint (which was not certified by peer review) is the author/funder. It is made available under a CC-BY 4.0 International license.

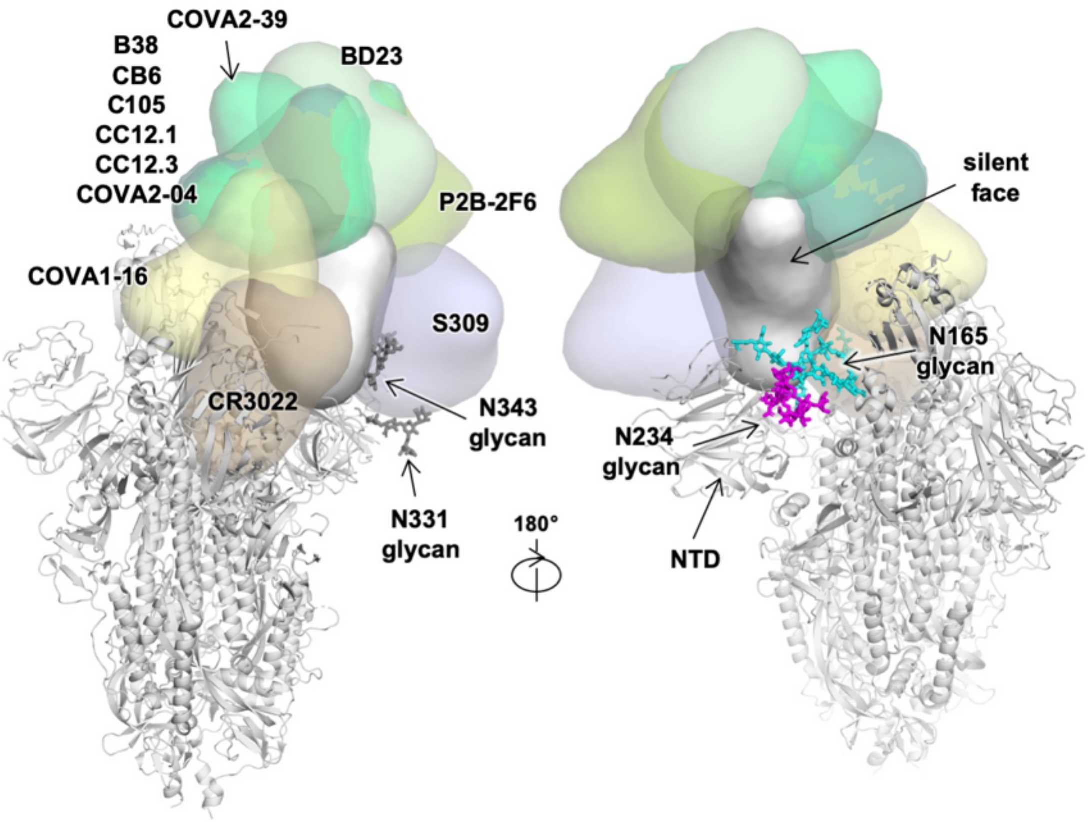

Supplementary Figure 11. The N-glycan on the N-terminal domain (NTD) also shields part of the RBD. The antibody-bound RBD, which is displayed and colored as in Figure 5, is shown in the up conformation on the S protein (PDB 6VSB) [5]. N-glycans on N165 (NTD), N234, N331, and N343 (RBD) are modelled according to the main glycoform observed at these sites in [6], and shown in stick representation. Antibody Fabs from published crystal and cryo-EM structures are represented as globular outlines in different colors as outlined in Figure 5. B38, CB6, C105, CC12.1, CC12.3, COVA2-04, COVA2-39, BD23, P2B-2F6 all bind at or around the receptor binding site. S309 binds to the elongated accessible face of the RBD in both up and down conformations, and CR3022 binds to the opposite face that is exposed in the RBD up conformation, but buried in the RBD down conformation.

bioRxiv preprint doi: https://doi.org/10.1101/2020.08.02.233536. this version posted August 3, 2020. The copyright holder for this preprint (which was not certified by peer review) is the author/funder. It is made available under a CC-BY 4.0 International license.

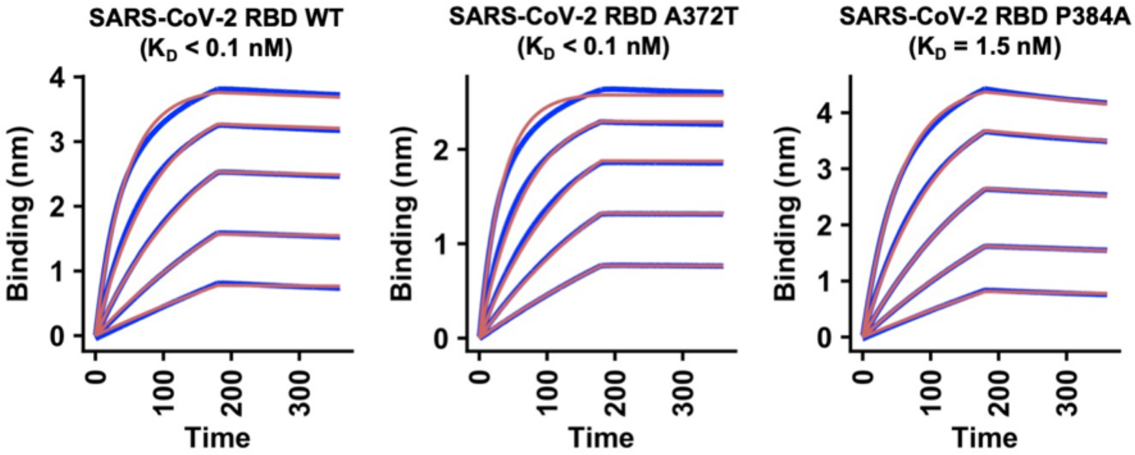

Supplementary Figure 12. Sensorgrams for binding of COVA1-16 IgG to SARS-CoV-2 RBD WT or mutants. Binding kinetics of COVA1-16 IgG to SARS-CoV-2 RBD WT, A372T, and P384A were measured by biolayer interferometry (BLI) with RBD on the biosensor and antibody in solution. Y-axis represents the response. Dissociation constants (K_D) for Fabs were obtained using a 1:1 binding model, which are represented by the red lines. Representative results of two replicates for each experiment are shown. A372T and P384A are the only two mutations that differ between the SARS-CoV-2 and SARS-CoV sequences in COVA1-16 epitope. The affinity of COVA1-16 IgG to the A372T mutant did not show any detectable difference from WT. Although the affinity (K_D) of COVA1-16 IgG to the P384A mutant decreases, the binding is still 100 times tighter than that measured between COVA1-16 IgG and SARS-CoV RBD (Figure S4B). As a result, the binding affinity of COVA1-16 to the RBD may be influenced by residues outside of the epitope as well as the dynamics of the RBD fluctuations between up and down conformations.

bioRxiv preprint doi: https://doi.org/10.1101/2020.08.02.233536. this version posted August 3, 2020. The copyright holder for this preprint (which was not certified by peer review) is the author/funder. It is made available under a CC-BY 4.0 International license.

Supplementary Table 1. X-ray data collection and refinement statistics

**Data collection**		
	COVA1-16 Fab + SARS-CoV-2 RBD	COVA1-16 Fab
Beamline	SSRL 12-1	SSRL 12-1
Wavelength (Å)	0.97946	0.97946
Space group	$P\,1\,2_1\,1$	$P\,4_1\,3\,2$
Unit cell parameters		
a, b, c (Å)	57.4, 124.9, 57.6	156.3, 156.3, 156.3
α, β, γ (°)	90, 96.1, 90	90, 90, 90
Resolution (Å) [a]	50.0-2.89 (2.95-2.89)	50.0-2.53 (2.58-2.53)
Unique reflections [a]	17,656 (845)	22,357 (1,084)
Redundancy [a]	3.7 (3.2)	37.0 (14.1)
Completeness (%) [a]	97.9 (93.9)	100.0 (100.0)
$\langle I/\sigma_I \rangle$ [a]	7.4 (1.2)	21.5 (1.3)
R_{sym} [b] (%) [a]	15.3 (69.1)	23.6 (>100)
R_{pim} [b] (%) [a]	9.0 (42.9)	3.8 (54.3)
$CC_{1/2}$ [c] (%) [a]	96.3 (66.8)	99.6 (52.1)
**Refinement statistics**		
Resolution (Å)	42.8-2.89	34.1-2.53
Reflections (work)	17,632	21,872
Reflections (test)	948	1,069
R_{cryst} [d] / R_{free} [e] (%)	23.7/29.4	21.2/24.4
No. of atoms	4,873	3,284
Macromolecules	4,845	3,223
Glycans	28	-
Average *B*-values (Å^2)	49	43
Macromolecules	49	43
Fab	45	43
RBD	56	-
Glycans	89	-
Wilson *B*-value (Å^2)	43	40
**RMSD from ideal geometry**		
Bond length (Å)	0.004	0.007
Bond angle (°)	0.74	1.02
**Ramachandran statistics (%)** [f]		
Favored	95.9	96.7
Outliers	0.16	0.0
**PDB code**	pending	pending

[a] Numbers in parentheses refer to the highest resolution shell.
[b] $R_{sym} = \Sigma_{hkl} \Sigma_i \mid I_{hkl,i} - \langle I_{hkl} \rangle \mid / \Sigma_{hkl} \Sigma_i I_{hkl,i}$ and $R_{pim} = \Sigma_{hkl} (1/(n-1))^{1/2} \Sigma_i \mid I_{hkl,i} - \langle I_{hkl} \rangle \mid / \Sigma_{hkl} \Sigma_i I_{hkl,i}$, where $I_{hkl,i}$ is the scaled intensity of the ith measurement of reflection h, k, l, $\langle I_{hkl} \rangle$ is the average intensity for that reflection, and *n* is the redundancy.
[c] $CC_{1/2}$ = Pearson correlation coefficient between two random half datasets.
[d] $R_{cryst} = \Sigma_{hkl} \mid F_o - F_c \mid / \Sigma_{hkl} \mid F_o \mid \times 100$, where F_o and F_c are the observed and calculated structure factors, respectively.
[e] R_{free} was calculated as for R_{cryst}, but on a test set comprising 5% of the data excluded from refinement.
[f] From MolProbity [7].

bioRxiv preprint doi: https://doi.org/10.1101/2020.08.02.233536. this version posted August 3, 2020. The copyright holder for this preprint (which was not certified by peer review) is the author/funder. It is made available under a CC-BY 4.0 International license.

Supplementary Table 2. Hydrogen bonds identified in the antibody-RBD interface using the PISA program

COVA1-16 Fab	Distance [Å]	SARS-CoV-2 RBD
H:ARG100b[NH2]	3.3	A:TYR369[O]
H:ARG100b[NE]	3.9	A:SER371[O]
H:ARG100b[N]	3.8	A:PHE377[O]
H:TYR100[N]	2.6	A:CYS379[O]
H:GLN101[NE2]	3.1	A:GLN414[OE1]
H:ARG97[NH1]	2.5	A:ASP427[O]
H:TYR32[OH]	3.1	A:ASP427[OD1]
H:THR28[N]	3.2	A:ASP427[OD2]
H:ARG97[NH1]	3.0	A:PHE429[O]
H:TYR100[O]	2.9	A:CYS379[N]
H:SER100c[O]	3.3	A:THR385[OG1]
H:GLN101[OE1]	3.8	A:GLN414[NE2]
L:ASN53[OD1]	3.2	A:ARG408[NH2]
L:LEU54[O]	3.7	A:ARG408[NE]

bioRxiv preprint doi: https://doi.org/10.1101/2020.08.02.233536. this version posted August 3, 2020. The copyright holder for this preprint (which was not certified by peer review) is the author/funder. It is made available under a CC-BY 4.0 International license.

SUPPLEMENTARY REFERENCES

1. Huo J, Zhao Y, Ren J, Zhou D, Duyvesteyn HME, Ginn HM, et al. Neutralization of SARS-CoV-2 by destruction of the prefusion spike. Cell Host Microbe. 2020. Epub 2020/06/26. doi: 10.1016/j.chom.2020.06.010. PubMed PMID: 32585135; PubMed Central PMCID: PMCPMC7303615.

2. Pinto D, Park YJ, Beltramello M, Walls AC, Tortorici MA, Bianchi S, et al. Cross-neutralization of SARS-CoV-2 by a human monoclonal SARS-CoV antibody. Nature. 2020. Epub 2020/05/19. doi: 10.1038/s41586-020-2349-y. PubMed PMID: 32422645.

3. Walls AC, Park YJ, Tortorici MA, Wall A, McGuire AT, Veesler D. Structure, function, and antigenicity of the SARS-CoV-2 spike glycoprotein. Cell. 2020;181:281-92.e6. Epub 2020/03/11. doi: 10.1016/j.cell.2020.02.058. PubMed PMID: 32155444.

4. Lawrence MC, Colman PM. Shape complementarity at protein/protein interfaces. J Mol Biol. 1993;234(4):946-50. Epub 1993/12/20. doi: 10.1006/jmbi.1993.1648. PubMed PMID: 8263940.

5. Wrapp D, Wang N, Corbett KS, Goldsmith JA, Hsieh CL, Abiona O, et al. Cryo-EM structure of the 2019-nCoV spike in the prefusion conformation. Science. 2020;367:1260-3. Epub 2020/02/23. doi: 10.1126/science.abb2507. PubMed PMID: 32075877.

6. Watanabe Y, Allen JD, Wrapp D, McLellan JS, Crispin M. Site-specific glycan analysis of the SARS-CoV-2 spike. Science. 2020;369:330-3. Epub 2020/05/06. doi: 10.1126/science.abb9983. PubMed PMID: 32366695; PubMed Central PMCID: PMCPMC7199903.

7. Chen VB, Arendall WB, 3rd, Headd JJ, Keedy DA, Immormino RM, Kapral GJ, et al. MolProbity: all-atom structure validation for macromolecular crystallography. Acta Crystallogr D Biol Crystallogr. 2010;66(Pt 1):12-21. doi: 10.1107/S0907444909042073. PubMed PMID: 20057044; PubMed Central PMCID: PMCPMC2803126.
